# Endoplasmic reticulum stress contributes to the decline in doublecortin expression in the immature neurons of mice with long-term obesity

**DOI:** 10.1038/s41598-022-05012-5

**Published:** 2022-01-19

**Authors:** Kiyomi Nakagawa, Saiful Islam, Masashi Ueda, Toshiyuki Nakagawa

**Affiliations:** 1 Department of Neurobiology, Gifu University Graduate School of Medicine, Gifu, 501-1194 Japan; 2 Bangladesh Council of Scientific and Industrial Research (BCSIR), Chattogram Laboratories, Chattogram, 4220 Bangladesh; 3 Department of Mental Retardation and Birth Defect Research, National Center of Neurology and Psychiatry, Tokyo, 187-8502 Japan

**Keywords:** Neuroscience, Alzheimer's disease, Dementia

## Abstract

Adult hippocampal neurogenesis (AHN) plays an important role in hippocampus-dependent function. The number of doublecortin (Dcx)-positive immature neurons in the dentate gyrus decreases over time, especially in the early stages of Alzheimer’s disease (AD), and is further reduced in later stages of AD. Obesity in midlife is associated with dementia later in life; however, the underlying mechanisms by which obesity results in the development of dementia later in life remain unknown. Here, we show that endoplasmic reticulum (ER) stress was activated in the hippocampus and processes of Dcx-expressing immature neurons were shortened, coexpressing CHOP in APP23 AD model mice with high-fat diet-induced long-term obesity and in aged *Lepr*^*db*/*db*^ (*db*/*db*) mice. Moreover, in cells differentiating from hippocampal neurospheres, *Dcx* mRNA was rapidly degraded via a microRNA (miRNA) pathway after thapsigargin treatment in vitro. These results indicate that loss of *Dcx* mRNA induced by ER stress during AHN may cause memory impairment in obese individuals later in life.

## Introduction

New neurons are generated in distinct regions of the adult human brain, i.e., the hippocampal subgranular zone (SGZ) and subventricular zone (SVZ)^1^. A total of 700 new neurons are generated daily in the hippocampus, but this number declines during aging^2^. Immature neurons in the dentate gyrus (DG) were not detected beyond adolescence in available human hippocampi^3,4^. However, under physiological conditions, adult hippocampal neurogenesis (AHN) was recently shown to be involved in hippocampus-dependent functions such as pattern separation^5^ and stress resilience^6^, and AHN is impaired in patients with Alzheimer’s disease (AD)^7^ and depression^8^. The number of cells positive for doublecortin (Dcx), which is specifically expressed in immature neurons, in the DG is decreased in patients with mild cognitive impairment (MCI)^9^ and those in the early stages of AD and is further reduced in later stages^7^. Staining for markers of various differentiation stages has revealed substantial impairment of Dcx-positive cell maturation in the DG of subjects with AD^7^ . Dcx, which stabilizes and maintains microtubules in maturing neurons^10^, has been identified as a causal gene of X-linked lissencephaly and double cortex syndrome, resulting in the arrest of neuronal migration^11,12^. The *Dcx* transcript has a long 3′ untranslated region (UTR), indicating its involvement in the regulation of mRNA stability^11^ and suggesting that *Dcx* mRNA is a target of microRNA (miRNA)-128^13^. Consistently, the *Dcx* transcript level is increased in *Dicer*^-/-^ neural stem cells (NSCs)^14^. Female mice heterozygous for a mutation in *Dcx* show abnormalities in hippocampal lamination, defects in contextual and conditioned fear memory, and mild deficits in Morris water maze (MWM) performance^15^, indicating that Dcx expression in the hippocampus is important for learning.

The number of people living with dementia is increasing; however, age-specific incidence rates of dementia have recently decreased in several countries because of decreases in the prevalence of some risk factors due to lifestyle changes. Twelve modifiable risk factors are known, including obesity, and eliminating these risk factors could prevent or delay the onset of dementia^16^. Consistently, the memory abilities of AD model mice with high-fat diet (HFD)-induced obesity^17^ and of the offspring of AD model mice crossed with diabetic *ob/ob* mice^18^ or diabetic *db/db* mice^19^ are impaired compared with that of AD model mice. A 28-year study on changes in body mass index (BMI) revealed that obesity at age 50 is associated with dementia^20^, indicating that obesity in midlife is related to dementia later in life. However, the underlying mechanisms by which obesity results in the development of dementia later in life remain unknown. Interestingly, the survival and proliferation of hypothalamic NSCs are disrupted by IκB kinase β (IKKβ)/nuclear factor-κB (NF-κB) activation in the context of long-term HFD-induced obesity^21^, and a decline in the release of exosomal miRNA due to the loss of hypothalamic NSCs regulates aging^22^. Several studies have suggested that endoplasmic reticulum (ER) stress is critical for obesity-induced inflammation^23,24^. Analysis of the brain transcriptomes of mice with different *APOE* genotypes and previously published RNA sequencing (RNA-seq) data from the brains of human patients with AD and control subjects has shown that the expression of activating transcription factor 4 (ATF4), which is increased during the unfolded protein response (UPR), is significantly upregulated in the brains of AD patients^25^. The UPR is induced by ER stress through three different pathways, which are initiated by inositol-requiring protein-1 (IRE1), protein kinase RNA-activated (PKR)-like ER-localized translation initiation factor 2α (eIF2α) kinase (PERK), and ATF6^26^. IRE1 mediates nonconventional *X-box binding protein-1* (*XBP-1*) mRNA splicing and ER-localized mRNA degradation through a process called regulated IRE1-dependent decay (RIDD). PERK phosphorylates eIF2α to suppress general translation, and ATF4 is specifically translated. Prolonged ER stress induces cell death^27^ in several diseases, including neurodegenerative diseases and diabetes, possibly through IRE1 activation and C/EBP-homologous protein (CHOP) expression^28^. eIF2α is phosphorylated at residue Ser51 by four protein kinases, including PERK, general control nonderepressible-2 (GCN2) kinase, double-stranded PKR, and heme-regulated inhibitor kinase (HRI), under several conditions, such as ER stress, amino acid starvation, viral infection, and heme deficiency, through a process known as the integrated stress response (ISR). Several studies have indicated that eIF2α phosphorylation and ATF4 expression are important for memory formation^29,30^. We have shown that ER stress and autophagy impairment enhance γ-secretase activity to increase amyloid-β (Aβ) production^31^ through the binding of ATF4 to the regulatory region of the presenilin-1 (PS1) gene^32^. Downregulation of ATF4 expression by quercetin in mouse models of AD improves memory impairment^19^.

Diabetes affects AHN by increasing the levels of glucocorticoids and hyperglycemia^33^. However, whether long-lasting obesity affects AHN remains unclear. Therefore, we investigated whether ER stress is activated in the brains of aged mice with long-term obesity and memory impairment and affects neurogenesis. In this study, we found that ER stress was activated and that the processes of Dcx-expressing immature neurons were shortened in the hippocampi of mice with long-term obesity. Furthermore, *Dcx* mRNA expression rapidly decreased in differentiating NSCs after thapsigargin treatment. These results indicate that the loss of *Dcx* mRNA induced by ER stress during AHN may underlie the memory impairment in obese individuals later in life.

## Results

### Behavior to novel object and swimming capacity were compromised in AD model mice with long-term obesity and diabetes model mice

**Figure 1:**
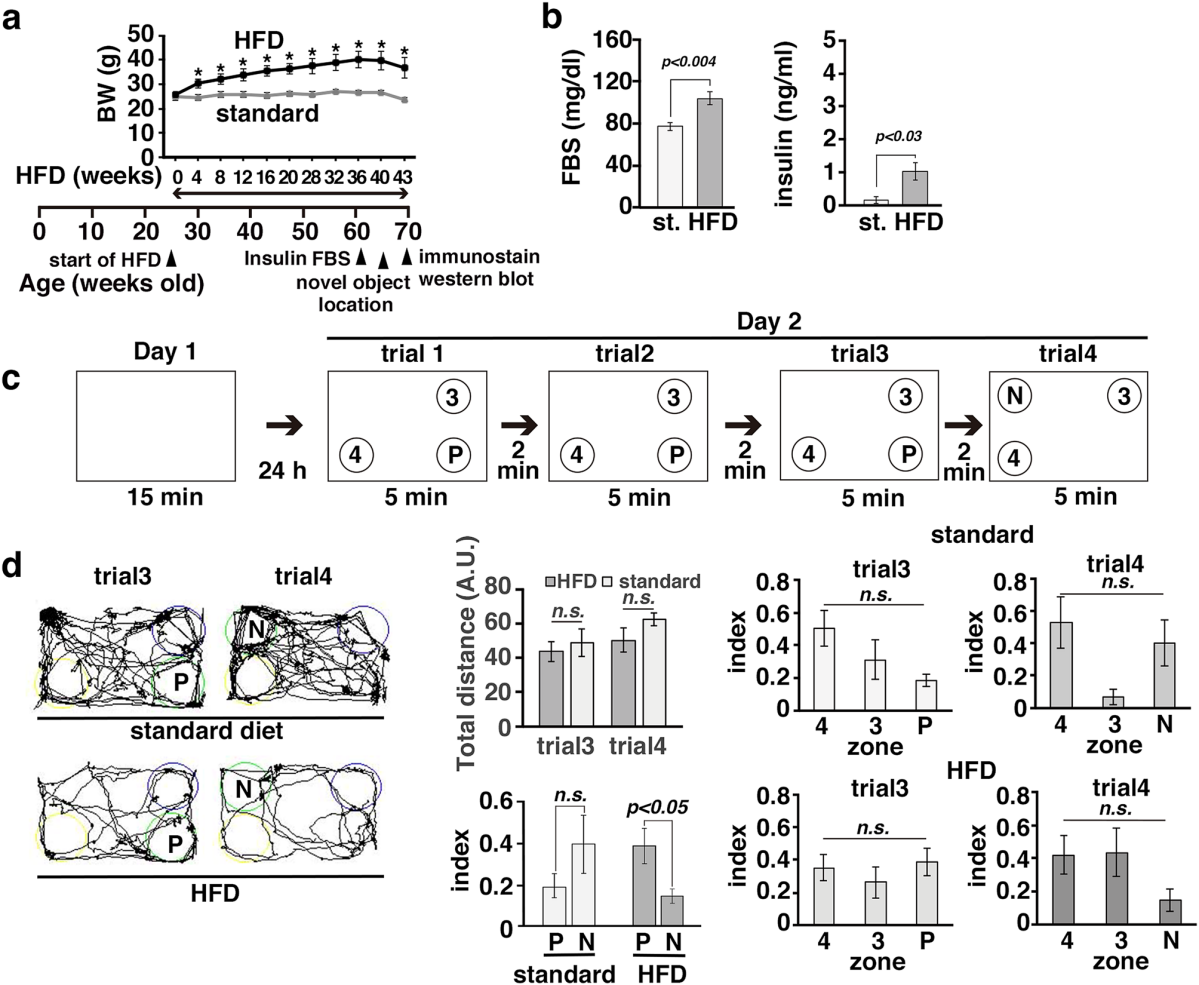
Memory was impaired in aged mice with long-term obesity. (**a**–**d**) APP23 mice (25 to 27 weeks old) were fed a standard chow diet (st) or a HFD (60% fat) for 43 weeks. The body weights (BWs) of APP23 (n = 7 standard diet-fed mice; n = 9 HFD-fed mice) (**a**) mice. The error bars represent the standard errors of the mean (SEMs). *p < *0.05*. Statistical significance (*p* < 0.05) was determined by Student’s t test. (**b**) Serum levels of FBS and insulin in standard diet-fed mice (n = 5) and HFD-fed mice (n = 8). Statistical significance (*p* < 0.05) was determined by Student’s t test. (**c**) Performances of mice with long-term obesity and control mice in the NOL test. One of three objects was moved from the previous location (P) to a novel location (N). (**d**) Representative trajectories of mice exploring the objects (left panels). The total distance traveled (arbitrary units: A. U.) and the exploration indices in each zone at trials 3 and 4 are shown. Statistical significance (*p* < 0.05) was determined by Student’s t test (total distance; index comparing between zones P and N) or one-way ANOVA followed by the Bonferroni post-hoc test [standard diet-fed APP23 mice: F(2,12) = 2.76, *p* = 0.10 (trial3), F(2,12) = 3.62, *p* = 0.06 (trial4); HFD-fed APP23 mice: F(2,24) = 0.54, *p* = 0.59 (trial3), F(2,24) = 2.07, *p* = 0.15 (trial4)]. Standard diet-fed mice (n = 5); HFD-fed mice (n = 9).

We previously showed that the expression levels of ATF4 are increased in the cortex, hippocampus, and amygdala in the offspring of obese and diabetic APP23 AD model mice crossed with *db/db* mice^19^ through a process known as the ISR^34^. ATF4 expression in the brain is decreased by quercetin, and this change is accompanied by an improvement in memory^19^. To examine the effect of ER stress on AHN in the brains of mice with long-term obesity, we first examined whether memory was impaired in HFD-induced obese mice. Obesity was induced by HFD feeding for 41 weeks in 66-week-old APP23 AD model mice (Fig. 1a–d) and for 64 weeks in 74-week-old wild-type C57BL6 mice (Suppl. Fig. 2). The serum levels of fasting blood sugar (FBS) and insulin were increased in both groups of mice subjected to long-term HFD feeding compared with mice fed a standard diet (Fig. 1b and Suppl. Fig. 2b). To examine whether long-term HFD-induced obesity affects memory, we performed the novel object location (NOL) test. Analysis of the ratio of the time spent exploring the target object to the time spent exploring all three objects revealed that the cognitive capacity of APP23 mice with long-term obesity was not significantly different from that of APP23 mice fed a standard diet. However, the time spent exploring the object at the novel location was significantly decreased in APP23 mice with long-term obesity, as they potentially exhibited neophobic behavior (Fig. 1c and d). Next, we examined whether spatial memory was affected in eighteen-week-old *Lepr*^*db*/*db*^ (*db*/*db*) mice, which had a higher body weight than wild-type and heterozygous littermates (control), even at 6 weeks of age (Suppl. Fig. 1a). In the MWM test, the latency to find the invisible platform during the acquisition phase was not changed and the number of platform crossings made by *db/db* mice was significantly decreased; these parameters were considerably affected by the differences in swimming speed and distance (Suppl. Fig. 1b). We next assessed aged wild-type mice with long-term obesity (Suppl. Fig. 2). The index for the object at the novel location was significantly increased in mice fed a standard diet but not in mice with long-term obesity (Suppl. Fig. 2e). These results suggested that long-term obesity affect spatial memory and behavior in mice.

### ER stress was activated in the brains of mice with long-term obesity

**Figure 2:**
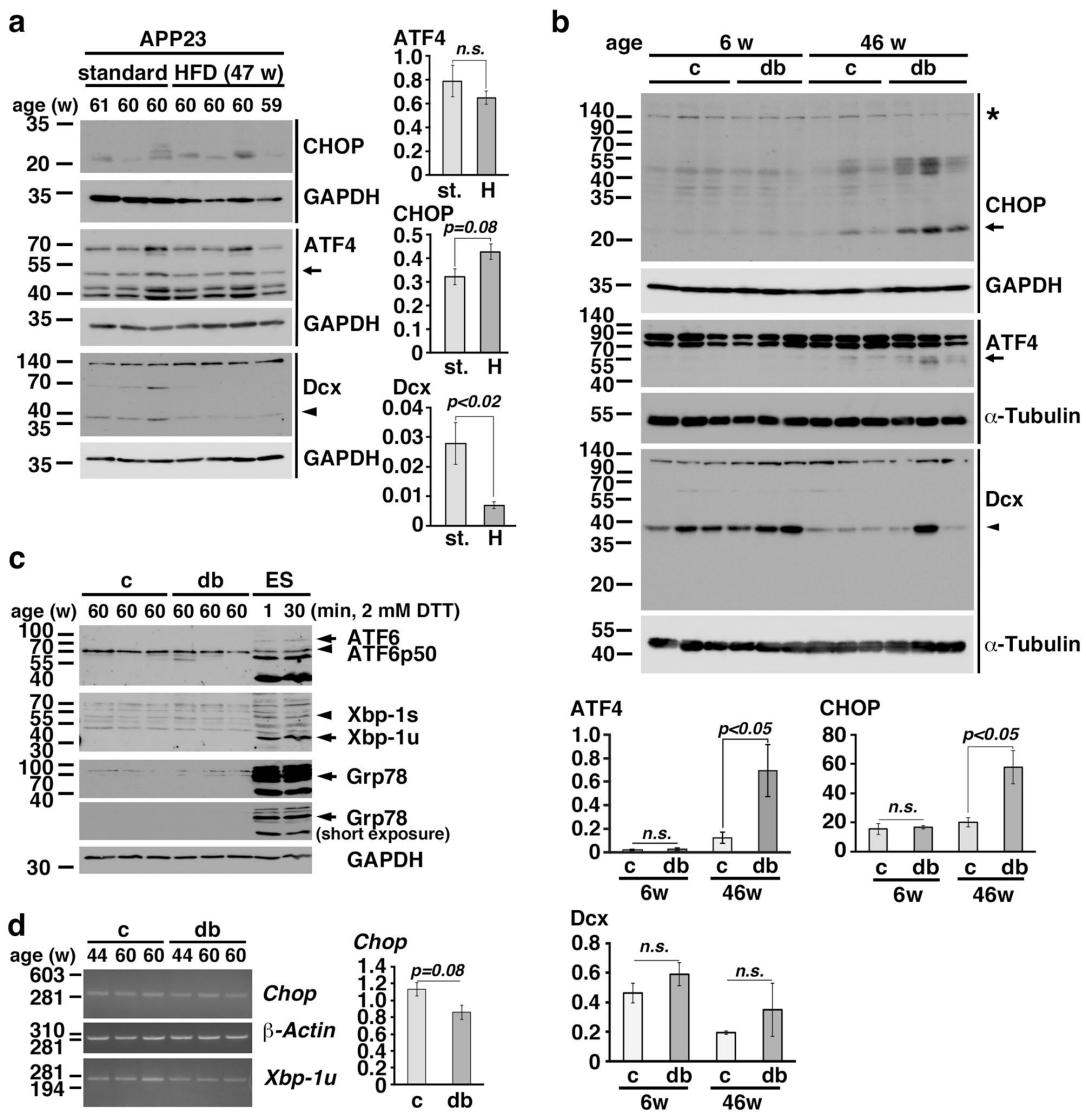
The ER stress was activated in the brains of mice with long-term obesity. (**a**, **b**) CHOP, ATF4, and Dcx expression in the hippocampi of APP23 mice with long-term HFD-induced obesity (**a**) and *db/db* mice (**b**). st.: standard diet; H: high-fat diet. The arrow in each panel indicates ATF4 or CHOP, and the arrowhead in each panel indicates Dcx. The asterisk indicates nonspecific signal (**b**). (**c**) ATF6 and Xbp-1 expression in the hippocampi of 60-week-old *db/db* mice. The arrowheads in each panel indicate the ATF6 fragment or Xbp-1 s. ES: mouse embryonic stem cell line. ES cells were treated with 2 mM DTT for 1 min or 30 min. (**d**) Semiquatitative RT–PCR of *Chop* in the hippocampi of aged control and *db*/*db* mice. (a-c) Statistical significance (*p* < 0.05) was determined by Student’s t test. The error bars represent the SEMs. n.s.: not significant.

To investigate the role of ER stress in the brains of mice with long-term obesity, we first used 59- to 61-week-old APP23 mice. After eight weeks of HFD feeding beginning at 21 weeks of age, the body weights of the mice fed a HFD were significantly increased. The body weights of the mice fed a HFD and the mice fed the standard diet were 42.5 ± 0.9 g and 28.3 ± 3.6 g, respectively, when the mice were approximately 60 weeks of age. Western blot analysis showed that the expression levels of CHOP in the hippocampi of APP23 mice with long-term obesity were increased compared with those in the hippocampi of mice fed the standard diet (Fig. 2a). After four weeks of HFD feeding, obesity was detected in wild-type C57BL/6 mice at the age of 16 weeks, and the body weights of mice fed a HFD and the standard diet were 55.8 ± 9.7 g and 36.0 ± 3.9 g, respectively, at the age of 86 weeks. Western blot analysis showed that the expression levels of CHOP in the hippocampi of C57BL6 mice with long-term obesity were significantly increased compared with those in the hippocampi of mice fed the standard diet (Suppl. Fig. 3). Furthermore, the expression levels of CHOP in the hippocampi of *db/db* mice with long-term obesity were significantly higher than those in the hippocampi of control mice at 46 weeks of age (Fig. 2b). We also observed an increase in ATF4 expression in wild-type and *db/db* mice with long-term obesity (Fig. 2b and Supp. Fig. 3). Since three signaling pathways, the PERK, ATF6, and IRE1 pathways, are activated during ER stress, we examined the expression of Xbp-1s and ATF6 fragmentation in the hippocampi of 60-week-old *db/db* mice. We found that the N-terminal cleavage product of ATF6 and Xbp-1s were not detected in the hippocampi of aged *db/db* mice but were detected in mouse ES cells treated with 2 mM dithiothreitol (DTT) (Fig. 2c). Therefore, we examined the levels of *Chop* mRNA in the hippocampi of aged control and *db*/*db* mice. We found that *Chop* mRNA levels were not increased in *db*/*db* mice (Fig. 2d), suggesting that ATF6 is not involved in CHOP induction in aged *db*/*db* mice. *Xbp-1s* expression was not observed in aged *db*/*db* mice. These results suggested that long-term obesity induced ER stress, particularly the ISR, in the hippocampus.

### CHOP and Dcx were coexpressed in the immature neurons of mice with long-term obesity

**Figure 3:**
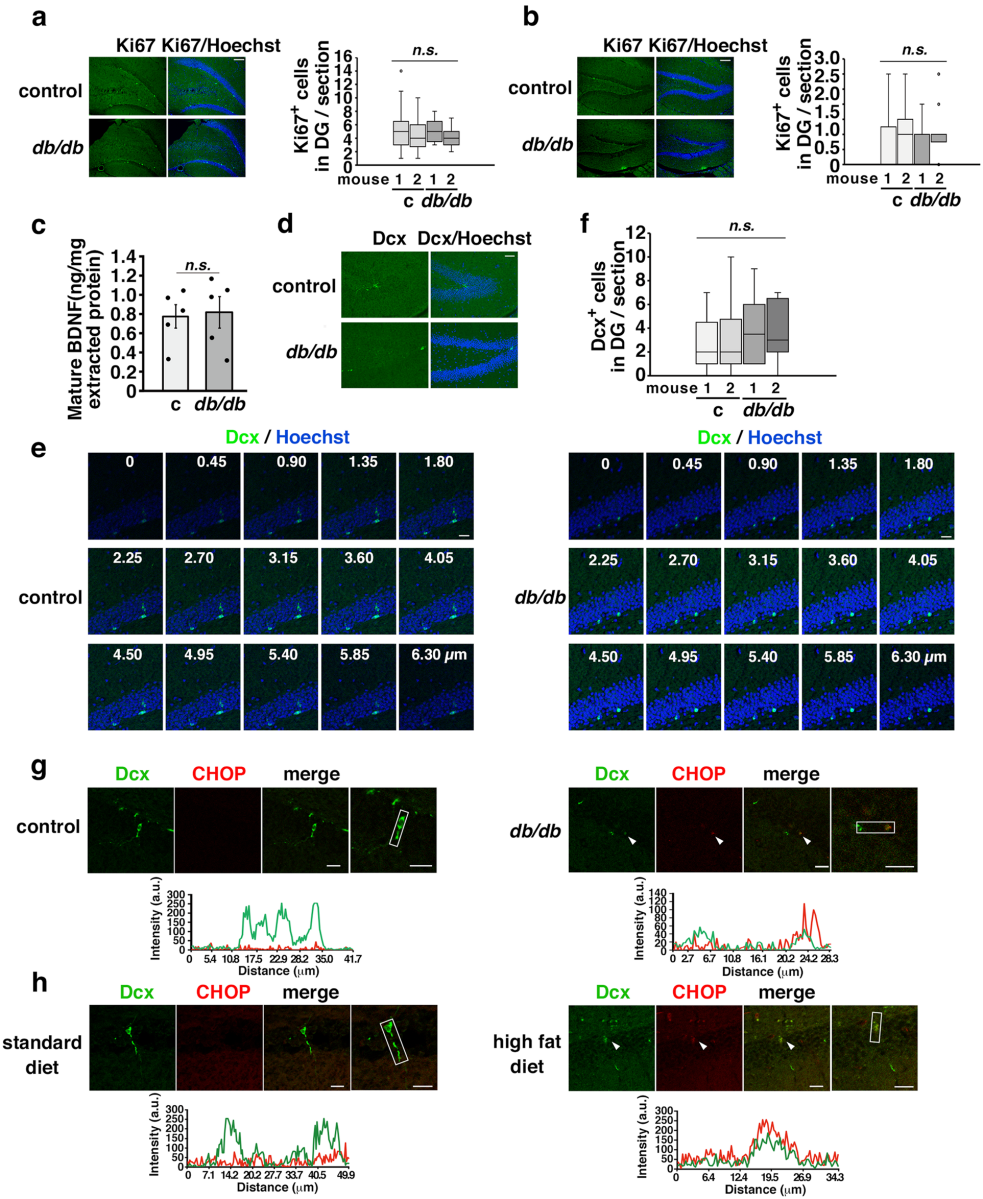
CHOP and Dcx were coexpressed in the immature neurons of mice with long-term obesity. (**a**, **b**) Ki67-positive ( +) cells in the SGZ of the DG/section in 10-week-old (c: control mice, n = 2; *db/db* mice, n = 2,) (**a**) and 45-week-old (control mice, n = 2; *db/db* mice, n = 2) (**b**) *db/db* mice. Scale bars: 100 µm. The box-and-whisker plot represents the number of cells per section from each animal. Statistical significance (*p* < 0.05) was determined by one-way ANOVA followed by the Bonferroni post-hoc test. (**c**) Mature BDNF expression in the hippocampi of 46-week-old mice. Control mice (n = 5); *db/db* mice (n = 5). Statistical significance (*p* < 0.05) was determined by Student’s t test. (**d–f**) Number of Dcx^+^ cells in the DG/section in 45-week-old control and *db/db* mice. Serial sections of the DG from zero to 6.30 µm imaged by confocal microscopy. Scale bars: 40 µm (**d**); 20 µm (**e**). The box-and-whisker plot represents the number of cell per section from each animal. Control mice (n = 2); *db/db* mice (n = 2) (**f**). Statistical significance (*p* < 0.05) was determined by one-way ANOVA followed by the Bonferroni post-hoc test. (**g**, **h**) CHOP was expressed in the Dcx-expressing neurons in 45-week-old *db/db* mice (**g**) and 69- to 71-week-old APP23 mice with long-term HFD-induced obesity (h). (**g**) Control mice (n = 2, 12 brain slices); *db/db* mice (n = 2, 12 brain slices). (**h**) Standard diet APP23 mice (n = 2, 8 brain slices); high fat diet APP23 mice (n = 3, 12 brain slices). The arrowheads in each panel indicate Dcx- and CHOP-positive cells. Scale bars: 20 µm. The fluorescence intensity profiles are shown in the square boxes (bottom panels).

To investigate the impact of the ER stress on AHN, we performed immunostaining for Ki67, which is continuously produced from the S phase until the cell cycle exit^35^, in 10-week-old (Fig. 3a) and 45-week-old (Fig. 3b) *db/db* mice using coronal plane brain sections at a distance of −1.5 to −2.5 mm from bregma. The number of Ki67-positive cells in the hippocampal DGs of *db/db* mice was similar to that in the DGs of control mice in both the young and aged groups (Fig. 3a, b). Additionally, the levels of mature BDNF in the hippocampi of 46-week-old control and *db/db* mice were similarly measured (Fig. 3c). The levels of Dcx in APP23 mice fed a HFD were significantly decreased compared with those in APP23 mice fed a standard diet (Fig. 2a), but the levels of Dcx did not differ between C57BL6 mice fed a HFD and C57BL6 mice fed a standard diet or between *db/db* mice and control mice (Fig. 2b and Suppl. Fig. 3). Therefore, we performed immunohistochemistry to examine the expression of Dcx in the hippocampus. Since Dcx is sensitive to postmortem breakdown^36^, the mice were anesthetized and quickly perfused with 4% paraformaldehyde for fixation, and then the expression of Dcx in the hippocampi of control and *db/db* mice was then examined by immunohistochemistry. The number of Dcx-positive cells in the DG did not differ between 45-week-old control and *db/db* mice (Fig. 3d, f) or between 10-week-old control and *db/db* mice (data not shown). We observed reduction of Dcx expression in processes in z-stacks by confocal microscopy of Dcx-positive cells in the DG in 45-week-old *db/db* mice (Fig. 3e). Using the method described by Plumpe et al.for the characterization of Dcx-positive cells^37^, more cells with short processes were observed in 45-week-old db/db mice than in 45-week-old control mice (Supplementary Fig. 4). These Dcx-positive cells in 45-week-old *db/db* mice expressed CHOP (Fig. 3g). Consistently, we observed that the Dcx-positive cells of APP23 mice with long-term obesity expressed CHOP (Fig. 3h). These results suggested that the ER stress was activated in the Dcx-positive immature neurons of mice with long-term obesity.

### Thapsigargin reduced Dcx expression in immature neurons through Dcx mRNA degradation

**Figure 4:**
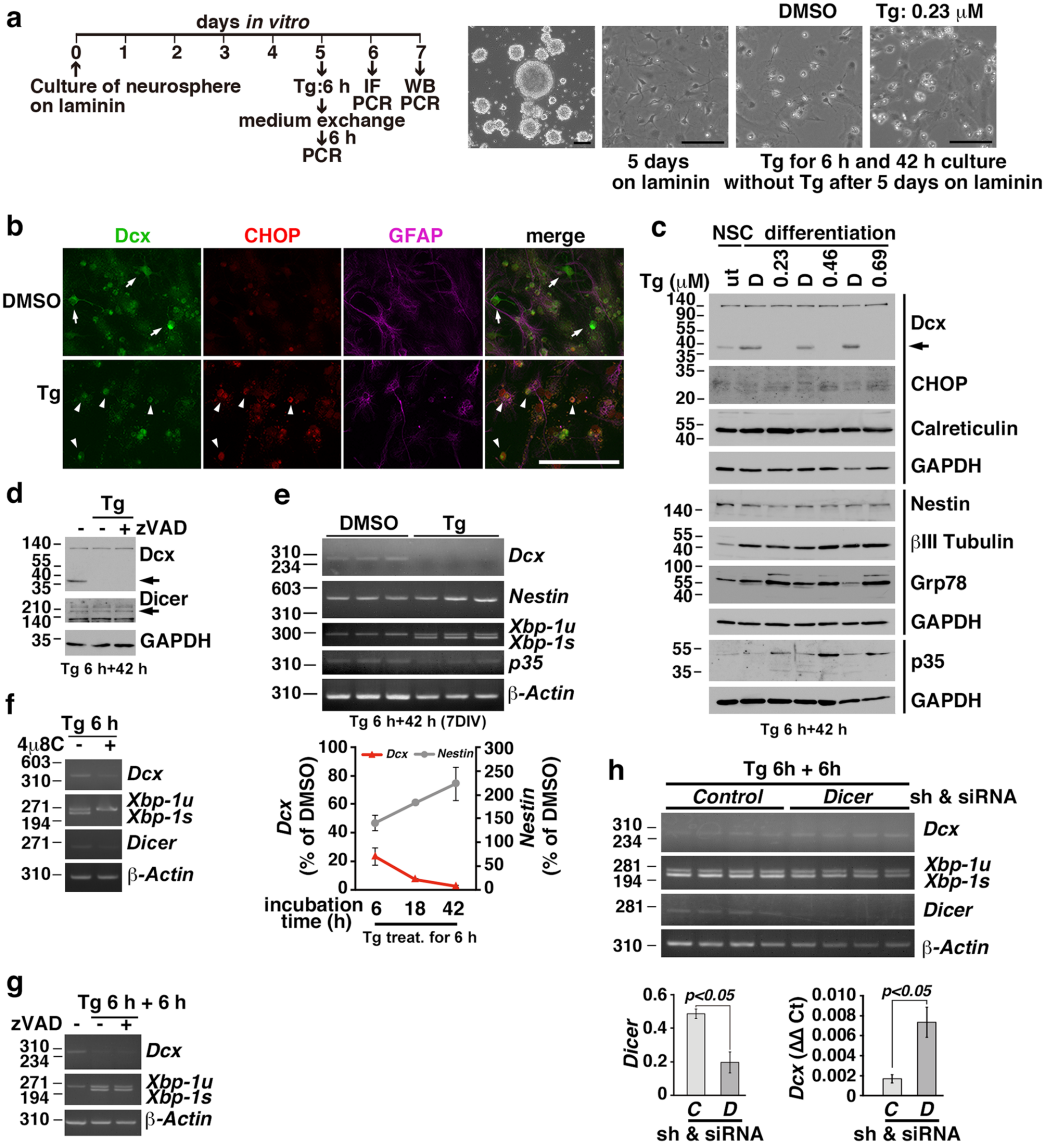
Thapsigargin reduced Dcx expression in immature neurons by decreasing the Dcx mRNA levels. (a) Experimental schedule and representative images. Hippocampal neurospheres from mice were differentiated on laminin for 5 days. The cells were treated with thapsigargin (Tg) for 6 h, washed once with medium, and then cultured in medium without Tg for the indicated amounts of times. (**b**) Immunostaining for Dcx, CHOP, and GFAP in differentiating cells cultured on laminin after Tg treatment for 6 h and in the absence of Tg for 18 h. Arrows: Dcx-expressing cells; arrowheads: Dcx- and CHOP-expressing cells. Scale bars: 100 µm (**a**, **b**). (**c**) Several neuronal lineage marker proteins, i.e., Dcx, calreticulin, nestin, βIII tubulin, were expressed in differentiating NSCs cultured on laminin, and the expression of the ER stress markers Grp78 and CHOP was induced by Tg. The arrow indicates Dcx. Cells were lysed at 42 h after Tg treatment for 6 h (Tg 6 h + 42 h). D: DMSO. (**d**) The cells were pretreated with 40 μM zVAD for 30 min, after which Tg (0.23 μM) was added and the cells were incubated for 6 h. After the medium was changed, the cells were incubated with 40 μM zVAD without Tg for 42 h. (**e**) Semiquantitative RT–PCR at 42 h after treatment with Tg (0.23 μM) for 6 h (upper panels). The mRNA levels of *Dcx* in cells cultured in medium without Tg were decreased at the indicated times after treatment with 0.23 μM Tg for 6 h (lower panel). The error bars represent the SEMs of 3 independent experiments. (**f, g**) The mRNA levels of *Dcx* were not changed after treatment with Tg (0.23 μM) for 6 h in the presence of the IRE1 inhibitor 4µ8C (**f**) or at 6 h after 0.23 μM of Tg treatment for 6 h in the presence of 40 μM zVAD (**g**). (**h**) Semiquantitative RT–PCR analysis of differentiating cells at 6 h after 0.23 μM Tg treatment for 6 h after the knockdown of *Dicer* using an shRNA and an siRNA (upper panels). Semiquantitative RT–PCR analysis of *Dicer* (lower left panel, *p* < *0.05,* Student’s t test). shRNA and siRNA for the *Control* (*C*) and for *Dicer* (*D*). Real-time RT–PCR analysis of *Dcx* (lower right panel, *p* < *0.05,* Student’s t test). The error bars represent the SEMs.

To investigate the effect of the ER stress on the Dcx expression in immature neurons, we cultured neurospheres isolated from the mouse hippocampus according to a previously established protocol^38^. After neurospheres were cultured on laminin-coated dishes for 5 days in vitro (DIV) (Fig. 4a), several marker proteins, i.e., Dcx, calreticulin, Nestin, and βIII tubulin, were expressed (Fig. 4c). Therefore, we challenged these cells with thapsigargin, a sarcoplasmic reticulum/ER Ca^2+^-ATPase inhibitor^39^ , for 6 h and then cultured them in new medium to induce ER stress according to a previously published method^27^. The expression levels of Dcx corrected for GAPDH after thapsigargin treatment were much lower than those after DMSO treatment as determined by western blot (Fig. 4c); however, the differences were not significant as determined by Student’s t test (*p* = 0.17). Consistently, immunostaining revealed that the processes in cells expressing both Dcx and CHOP was shortened (Fig. 4b), as was observed in vivo (Fig. 3g, h). Interestingly, loss of Dcx protein expression was observed in thapsigargin-treated cells, but the levels of other marker proteins were not decreased (Fig. 4c). The proneural basic helix-loop-helix neurogenin 1 and 2 proteins directly activate *Dcx* expression by binding to the promoter and upregulating *p35* expression^40^. p35 protein expression was also not decreased by thapsigargin treatment (Fig. 4c). To examine the involvement of the ubiquitin–proteasome system, autophagy-lysosomal pathway, or apoptosis in the loss of Dcx protein expression in thapsigargin-treated cells, we added MG132, E-64d/pepstatin A, or z-VAD-FMK to immature neurons and then treated them with thapsigargin. Since the protein levels of Dcx were not rescued by 25 µM MG132, 10 µg/mL E-64d and 10 µg/mL pepstatin A (data not shown), or 40 µM z-VAD-FMK (Fig. 4d), as determined by western blotting, we measured the levels of *Dcx* mRNA by semiquantitative reverse transcriptase polymerase chain reaction (RT–PCR) (Fig. 4e). We found that the mRNA levels of *Dcx*, but not *Nestin* or *p35*, quickly decreased during incubation in new medium after thapsigargin treatment (lower panel in Fig. 4e). The levels of *Dcx* mRNA were not rescued by the IRE1 inhibitor 4µ8C, indicating that *Dcx* mRNA is not a substrate for RIDD (Fig. 4f). Since the *Dcx* transcript has a long 3′ UTR, which binds the Musashi1 RNA-binding protein, mRNA stability and translation are possibly regulated by the binding proteins^11^. We found that the levels of *Dcx* mRNA were not rescued by zVAD (Fig. 4g), suggesting that caspase substrates are not involved in *Dcx* mRNA stability and that apoptosis does not decrease *Dcx* mRNA expression. Next, we knocked down *Dicer* in differentiating NSCs using a small hairpin RNA (shRNA) and small interfering RNA (siRNA) and then treated the cells with thapsigargin to examine whether the ER stress-induced reduction in *Dcx* mRNA expression was regulated by Dicer, as *Dcx* mRNA expression has been reported to be upregulated in *Dicer*-deficient NSCs^14^. Neurospheres cultured on laminin were infected with lentiviral particles carrying an shRNA targeting *Dicer* at 1 DIV, transfected with an siRNA targeting *Dicer* at 4 DIV, and then treated with thapsigargin at 5 DIV. Real-time RT–PCR showed that the thapsigargin-induced downregulation of *Dcx* mRNA expression was significantly prevented by the knockdown of *Dicer* in differentiating NSCs (Fig. 4h).

**Figure 5:**
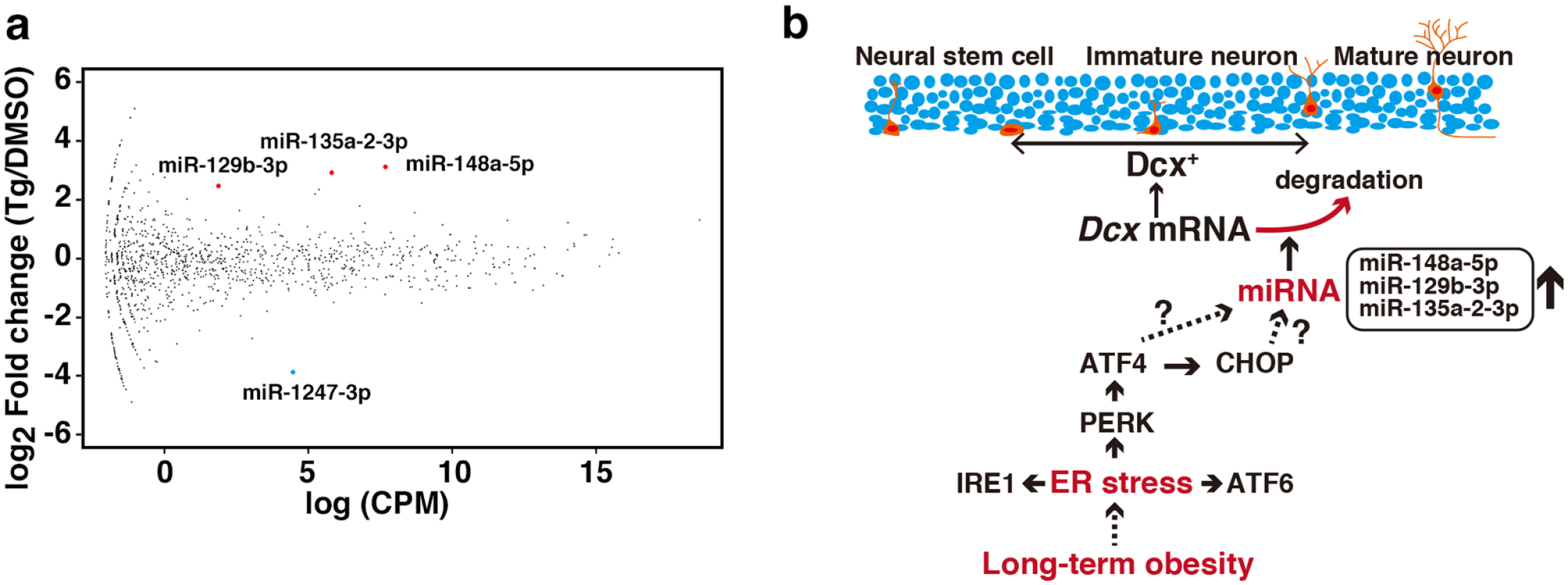
ER stress increased the miRNA expression in differentiating neurospheres. (**a**) Differential expression analysis of miRNAs in DMSO-treated cells (n = 3) and thapsigargin-treated cells (n = 3) was performed using edgeR. The y-axis indicates the expression ratio [log_2_ (fold change)], and the x-axis indicates the average of expression [log (CPM)]. Solid red circle: upregulated miRNA; solid blue circle: downregulated miRNA. FDR < 0.05. (**b**) Model of the effect of long-term obesity on neural maturation during AHN.

To identify the miRNAs upregulated during ER stress, we performed small RNA-seq on differentiating neurospheres. Analysis of the differential expression of *microRNAs* in thapsigargin treated immature neurons revealed that some *microRNA*, such as miR-148a-5p, miR-129b-3p, and miR-135a-2-3p, were significantly increased upon thapsigargin treatment (Fig. 5a).

## Discussion

ER stress is activated by several chronic diseases, including obesity, diabetes^41^, and depression^42^, which may cause dementia later in life^16^. However, it is unknown why it takes a long period of time for dementia to develop after body weight increases. In the present study, we demonstrated that long-term obesity induced ER stress, mainly via the ATF4-CHOP axis, in the hippocampal DG, leading to a decrease in the number of processes on Dcx-expressing immature neurons due specifically to the loss of *Dcx* mRNA stability.

The ATF4-CHOP axis is initiated by eIF2α phosphorylation, which is mediated by four protein kinases, through a process called the ISR, which contributes to the pathogenesis of diseases, including cognitive disorders^29^. For example, PERK phosphorylation^43^ and eIF2α phosphorylation^44^ are observable in AD patients. Consistently, the gene expression of the ISR-related signaling molecule ATF4 is upregulated in the AD brain^25^. Since ATF4 directly binds to the promoter of the *CHOP* (also known as growth arrest and DNA damage-inducible protein, *Gadd153*) gene^45^ and *CHOP* induction is dependent on ATF4 expression, CHOP is expressed when the ISR is activated. CHOP was detected in the hippocampi of mice with long-term obesity as determined by western blotting (Fig. 2), and immunohistochemistry and confocal microscopy confirmed that CHOP was localized in Dcx-expressing immature neurons of the hippocampal DG (Fig. 3g, h). We observed significantly short processes on Dcx-positive immature neurons in the hippocampal DGs of mice with long-term obesity (Fig. 3). Our experiment showed that the pan-caspase inhibitor zVAD had no effect on the protein expression of Dcx or on *Dcx* mRNA stability under the ER stress condition (Fig. 4d, g). Further experiments are needed to explore the mechanisms of dendritic loss in Dcx-expressing immature neurons. ER stress is involved in the pathogeneses of obesity and diabetes^46,47^, which exacerbate cognitive dysfunction in mouse model of AD crossing *db/db* mice^48^. We did not observe the cleavage product of ATF6 or XBP-1s in 20 µg hippocampal lysates even after a longer exposure as determined by western blot (Fig. 2c). Although *chop* expression is regulated by the PERK^49^ and ATF6^50^ pathways through the binding of ATF4^45^ and ATF6^51^ to the amino acid-response element (AARE) and ER stress response element (ERSE) of the *chop* promoter, respectively, *chop* mRNA levels were not increased, and *xbp-1s* mRNA was not observed in the hippocampi of aged *db/db* mice (Fig. 2d), suggesting that ATF6 and Xbp-1 are not involved. The results presented herein demonstrated the potential activation of ER stress/ISR in the immature neurons of mice with long-term obesity. However, the mechanisms of ER stress/ISR activation in immature neurons remain unclear. Reactive oxygen species (ROS) are produced as byproducts of protein oxidation in the ER through ER oxidoreductin-1 (ERO1) and protein disulfide isomerase (PDI)^52^. ERO1α transcription is increased by CHOP^53^ . Overexpression of ERO1β causes ER stress in pancreatic β cells^54^. ER stress is also elicited by ROS^55^ and by the inactivation of PDI through nitrosylation^56^. In the C17.2 NSC line, oxidative stress induced by high-glucose medium activates ER stress and prevents Tuj1 and glial fibrillary acidic protein (GFAP) expression, which is rescued by the superoxide dismutase mimetic tempol and ER stress inhibitor 4-phenylbutyrate^57^. Thus, reduction–oxidation (redox) reactions and ER stress stimulate each other^58^. Although we do not know which occurs first in the brains of obese mice, we hypothesize that oxidative stress induces ER stress/ISR in Dcx-expressing immature neurons in vivo. To confirm this hypothesis, further studies are needed; for example, in vivo dynamic nuclear polarization magnetic resonance imaging (DNP-MRI)^59^ may allow detection of redox conditions in the brains of young and aged obese mice.

Differentiating NSCs were treated with thapsigargin for 6 h, washed, and cultured in medium without thapsigargin. ER stress was activated in these cells, as indicated by the induction of 78-kDa glucose-regulated protein (Grp78)/immunoglobulin heavy chain-binding protein (BiP) and CHOP expression. The Dcx protein was absent in thapsigargin-treated cell lysates, although the proteins Nestin and βIII Tubulin were present (Fig. 4c). Interestingly, we found that loss of the Dcx protein induced by ER stress was mainly caused by the rapid elimination of *Dcx* mRNA (Fig. 4e) because the protein expression of Dcx was not rescued by inhibitors of the proteasome, autophagy, and caspase. Dcx is a microtubule-associated protein that leads to microtubule polymerization^60,61^, and its mRNA is not a substrate for RIDD (Fig. 4f), probably because *Dcx* mRNA is not an ER-localized mRNA. The *Dcx* transcript has a long 3′ UTR^11^, which binds Musashi1^62^ and miRNAs^13,14,63–66^ to repress *Dcx* mRNA translation or to regulate *Dcx* mRNA stability. The mRNA levels of *Dcx* were decreased in differentiating NSCs at 6 h after thapsigargin treatment for 6 h (Fig. 4e), indicating ER stress induced the loss of *Dcx* mRNA. The mRNA expression of *Dcx* in differentiating NSCs was significantly rescued by *Dicer* knockdown after thapsigargin treatment (Fig. 4h). Consistently, the *Dcx* transcript level is increased in unstressed adult NSCs from *Dicer* knockout mice^14^. The *Dcx* 3′ UTR is targeted by miR-128, resulting in the downregulation of Dcx protein expression in SH-SY5Y neuroblastoma cells^13^ and in Neuro2A cells^14^, but the mechanism by which *Dcx* mRNA is regulated is controversial^13,14^. Moreover, it may be interesting to investigate the serum levels of miR-128 in individuals with preclinical AD or MCI and long-term obesity since the circulatory levels of miR-128 are significantly increased in patients with type 2 diabetes and depression compared to those in patients with type 2 diabetes^67^. It has been suggested that *miRNA* biogenesis is regulated upon ER stress^68^. Emde et al. indicated that microRNA biogenesis is inhibited by thapsigargin through the inhibition of DICER activity in the hybrid motor neuron cell line^69^, in contrast, Behrman et al. demonstrated that thapsigargin induces *microRNA-708* to control rhodopsin expression in MEFs^70^, and Bartoszewski, R., et al. showed that tunicamycin induces *microRNA-346* to control TAP1 expression in MEFs^71^. We found that the expression levels of miR-148a-5p, miR-129b-3p, and miR-135a-2-3p were significantly increased [log_2_ fold change > 1, false discovery rate (FDR) < 0.05] in differentiating neurospheres under the condition of ER stress (Fig. 5a). These evidences and our results demonstrate that ER stress may stimulate a miRNA pathway to regulate *Dcx* mRNA stability, resulting in the loss of Dcx in the immature neurons of the hippocampal DG (Fig. 5b). Although *Dcx* is predicted to be a target of miR-129b-3p by miRDB^72^, more experiments are needed to explore the mechanism of *Dcx* mRNA degradation.

In conclusion, ER stress and the ISR are activated in the hippocampal DG immature neurons of mice with long-term obesity. Loss of *Dcx* mRNA by ER stress/ISR during AHN may underlie the memory impairment that occurs later in the lives of obese subjects.

## Methods

### Western blot analysis and chemicals

Western blotting was performed as described previously^32^. Mouse tissue and cells were lysed in RIPA [50 mM Tris–HCl (pH 7.5), 150 mM NaCl, 0.1% sodium dodecyl sulfate (SDS), 0.1% sodium deoxycholate, and 1% Nonidet P-40] with Roche cOmplete™ mini tablets (Roche, Mannheim, Germany), 25 µM MG132 (EMD Chemicals, Inc; San Diego, CA), and phosphatase inhibitors, [20 mM β-glycerophosphate (Sigma–Aldrich Co., LLC; St. Louis, MO) and 20 mM sodium orthovanadate (Fujifilm, Osaka, Japan)]. After centrifugation at 13,100 × g, the supernatants were used for western blotting with the following antibodies: anti-CHOP (Thermo Fisher Scientific, Waltham, MA; Cell Signaling Technology, Inc; Danvers, MA), anti-ATF4 (Santa Cruz Biotechnology, Inc; Santa Cruz, CA; Thermo Fisher Scientific), anti-p35 (C-19), anti-GAPDH (Santa Cruz Biotechnology, Inc), anti-α-tubulin (Sigma–Aldrich Co., LLC; St. Louis, MO, anti-Dcx (Abcam, Cambridge, UK; Novus Biologicals, Centennial, CO; Santa Cruz Biotechnology, Inc), anti-calreticulin (StressGen Biotechnologies Corp., Victoria, Canada), anti-Nestin (EMD Millipore Corp., Temecula, CA), anti-βIII tubulin (Covance, Inc; Princeton, NJ), and anti-BiP (Grp78, KDEL) (ENZO Life Sciences International, Inc., Plymouth Meeting, PA). HRP-conjugated anti-rat, anti-mouse, and anti-rabbit IgG (H + L) antibodies (SouthernBiotech; Birmingham, AL) and thapsigargin (Santa Cruz Biotechnology, Inc.) were also used. FBS levels were measured with a FreeStyle Freedom Lite device (Abbott Diabetes Care Inc., Alameda, CA). Insulin levels were measured by a mouse insulin enzyme-linked immunosorbent assay (ELISA) Kit (RTU) (Shibayagi, Gunma, Japan). BDNF levels were measured with a Mature BDNF *Rapid*™ ELISA Kit (Bisensis Pty Ltd., Thebarton, Australia). All other chemicals were purchased from Wako Pure Chemical Industries, Ltd. (Osaka, Japan), Kanto Chemical Co., Inc., (Tokyo, Japan), and Sigma–Aldrich Co., LLC.

### Animals and the HFD

C57BL/6J mice were purchased from Japan SLC, Inc. (Hamamatsu, Japan). APP23 mice, which express human *APP*_751_ cDNA and a Swedish double mutation on the C57BL/6 genetic background^73^, were kindly provided by Dr. M. Staufenbiel (Novartis Pharma Ltd; Basel, Switzerland). Obese and diabetic *db/db* (Lepr^*db/db*^) mice on the C57BL/6 genetic background were purchased from The Jackson Laboratory (Bar Harbor, ME). The mice were housed in a temperature- and light-controlled room (24 °C; 12-h light/dark cycle) and fed on AIN93G (standard diet) or HFD-60 (Oriental Yeast Co., Ltd; Tokyo, Japan). All animal studies were approved by the Gifu University Graduate School of Medicine Animal Care and Use Committee and were performed in accordance with the guidelines for experiments on animals provided by the Ministry of Education, Culture, Sports, Science and Technology of Japan. All animal experiments were performed according to the ARRIVE guidelines.

### Behavioral tests

The NOL test was performed according to the method described by Roy et al.^74^. Briefly, each mouse was habituated to a cage without objects for 15 min on day 1. The mice were exposed to three different objects, i.e., conical (diameter x height: 5 × 11.5 cm), cylindrical (6.5 × 10.5 cm), and reagent (5 × 13.2 cm) bottles, for 5 min three times at 2-min intervals on day 2. The mice were placed in their home cages for a retention interval (two minutes). Then, one of the objects was moved to the opposite corner. The behavior of each mouse was monitored using video recording software and an automated tracking system (SMART v3.0 software; Panlab, Barcelona, Spain) and a video camera (HDC-HS350; Panasonic; Osaka, Japan). Three zones (trial3: 3, 4, P; trial4: 3, 4, N) were set at a distance of 4 cm from the object in the far corners of the arena. The exploration index was calculated as the time spent exploring the object placed at the previous location (P), at a novel location (N), at a zone 3 or at a zone 4 divided by the total time spent in three zones [index P: P/(3 + 4 + P); index N: N/(3 + 4 + N); index 3: 3/(3 + 4 + P or N); index 4: 4/(3 + 4 + P or N)]. When the distance between the nose of the mouse and an object was less than 2 cm or when the mouse sniffed or touched the object with its snout, the mouse was considered to be exploring the object, as previously described^75^. The cages and objects were cleaned with 70% ethanol and 1% acetic acid solution before each trial to eliminate dominant odors.

The MWM test was performed according to a previously described protocol^76^. Briefly, the apparatus was a 100-cm diameter tank containing water at a temperature of approximately 22 °C water, skim milk and a submerged platform. Four acquisition trials from each of the five starting positions were performed each day. The time limit for each trial was 60 s. Mice that did not reach the platform were guided to the platform and left there for 30 s. On day 5, the platform was removed for the probe test. Each trial was recorded and analyzed with the SMART v3.0 automated tracking system.

### Immunostaining

Immunostaining was performed as described previously^27^ and analyzed by fluorescence microscopy (BZ-9000, Keyence; Osaka, Japan) and confocal microscopy (LSM710, Carl Zeiss; Göttingen, Germany). Briefly, mice were anesthetized and perfused with phosphate-buffered saline (PBS) and fixed with 4% paraformaldehyde in 0.1 M phosphate buffer (PB). The mouse brains were postfixed for 2 h in the same fixative, which was then replaced with 15% sucrose in 0.1 M PB. The brains were embedded into the optimal critical temperature (OCT) compound (Sakura Finetech USA, Inc., Torrance, CA), and 14-µm-thick sections (coronal plane at a distance of −1.5 to −2.5 mm from bregma: The Mouse Brain in Stereotaxic Coordinates, second edition, Academic Press) were obtained with a cryostat (HM 550, Carl Zeiss). The secttions (14 µm) were incubated in PBS supplemented with 10% normal goat serum (Jackson ImmunoResearch Laboratories, Inc., West Grove, PA) and 0.1% Triton X-100 at room temperature for 1 h. The sections were then incubated with an anti-Dcx antibody and an anti-CHOP antibody or an anti-Ki67 antibody (Thermo Fisher Scientific) in PBS containing 1% normal goat serum and 0.1% Triton X-100 at 4 °C for 12 h. For the detection of Ki67, the sections were incubated in 10 mM sodium citrate (pH 6) at 80 °C for 30 min and cooled to room temperature before the antibody was added. The sections were incubated with Alexa Fluor 488-conjugated anti-rabbit IgG (H + L) and Alexa Fluor 546-conjugated anti-mouse IgG (H + L) antibodies (Thermo Fisher Scientific) and Hoechst [1 µg/mL bis-benzimide (Sigma–Aldrich Co. LLC)] to detect fluorescence signals and nuclei. The fluorescence intensity profiles were analyzed with Zen software (Carl Zeiss). The count of Ki67-positiveand Dcx-positive cells were counted by two people using images obtained by fluorescence microscopy (BZ-9000) and confocal microscopy (LSM710), respectively.

### Cell culture and ER stress treatment

Neurospheres were cultured and isolated from the hippocampi of 10-day-old C57BL/6J mice according to a previously described protocol^38^. Neurospheres were grown in neurobasal medium supplemented with B-27 without vitamin A, 20 ng/mL basic FGF, 20 ng/mL EGF, GlutaMAX, and gentamicin (Thermo Fisher Scientific). For differentiation, the cells were plated on dishes coated with natural mouse laminin (Thermo Fisher Scientific); the next day, the medium was replaced with medium A, which was comprised of Dulbecco’s modified Eagle’s medium and Ham’s F-12 (DMEM/F12, Wako Pure Chemical Industries, Ltd.), MACS® NeuroBrew®-21 (Miltenyi Biotec, Bergisch Gladbach, Germany), 0.5 × N-2 supplement (Thermo Fisher Scientific), 20 ng/mL basic FGF, and gentamicin. On the following days, the medium A was changed in the morning and replaced with medium B, which consisted of DMEM/F12, MACS® NeuroBrew®-21, 0.5 × N-2 supplement, and gentamicin, in the afternoon. Then, the medium was changed every two days. The cells were maintained at 37 °C in an atmosphere containing 5% CO_2_. Thapsigargin was added to the medium at a final concentration of 0.23–0.69 µM, after which the cells were incubated for 6 h and washed with medium. The medium was replaced with new medium, and the cells were incubated for the indicated amounts of times. Knockdown of *Dicer* was induced by an shRNA-expressing lentivirus (sc-4090-V, Santa Cruz) and an siRNA (s101206, Thermo Fisher Scientific). Differentiating cells were infected with a lentivirus expressing an shRNA targeting *Dicer* or a control shRNA (sc-108080, Santa Cruz) with 5 µg/mL polybrene at 1 DIV and then transfected with an siRNA targeting *Dicer* or a control siRNAs at 4 DIV using TransIT-X2 (Takara Bio Inc., Shiga, Japan). The following day, the cells were treated with thapsigargin. The sequences of the siRNAs targeting *Dicer* were as follows: 5′-GCCGAUCUCUAAUUACGUAtt-3′ and 5′-UACGUAAUUAGAGAGAUCGGCgc-3′. For immunostaining, the cells were fixed with 4% paraformaldehyde in 0.1 M PB 4 °C for 15 min and then incubated in PBS containing 10% normal goat serum and 0.3% Triton X-100 at room temperature for 1 h. The cells were incubated with an anti-Dcx antibody, an anti-CHOP antibody and an anti-GFAP antibody (Thermo Fisher Scientific) in PBS containing 1% normal goat serum and 0.1% Triton X-100 at 4 °C for 12 h. The cells were then incubated with Alexa Fluor 488-conjugated anti-rabbit IgG (H + L) and Alexa Fluor 546-conjugated anti-mouse IgG (H + L) and Alexa Fluor 647-conjugated anti-rat IgG (H + L) antibodies (Thermo Fisher Scientific).

Mouse embryonic stem (ES) cells were grown in DMEM supplemented with 15% normal bovine serum, MEM nonessential amino acids (Thermo Fisher Scientific), 0.1 mM 2-mercaptoethanol (Sigma–Aldrich Co., LLC.), and 1 mM sodium pyruvate (Sigma–Aldrich Co., LLC.). Mouse ES cells were treated with 2 mM DTT for 1 or 30 min, and cells were then lysed by RIPA for western blot analysis.

### Small RNA sequencing

Total RNA was purified using a miRNeasy mini kit (QIAGEN, Hilden, Germany) from differentiating neurospheres cultured for 6 h after treatment with DMSO or 0.23 µM of thapsigargin for 6 h. Six small RNA-seq libraries (DMSO: n = 3; thapsigargin: n = 3) were generated from the purified total RNA using NEB Next® Multiplex Small RNA Library Prep Set for Illumina® (Set 1) (New England Biolabs, Ipswich, MA) according to the manufacturer’s instructions. Fifty bp single-end sequencing was performed with NovaSeq 6000 (Illumina Inc., San Diego, CA). After validating the quality of the raw sequencing reads was validated with FastQC (Ver.0.11.7), the raw sequencing reads were trimmed with Trimmomatic (Ver.0.38). Filtered sequencing data were mapped with STAR (Ver.2.7.4a), and count per million (CPM) values were normalized with featureCounts (Ver.1.6.3). Differential expression analyses of miRNAs in DMSO-treated cells and thapsigargin-treated cells were performed using edgeR (Ver.3.26.8).

### Semiquantitative and quantitative RT–PCR

RNA was isolated from cells with TRIzol (Thermo Fisher Scientific) as described previously^32^. Reverse transcription was performed using M-MLV reverse transcriptase (Thermo Fisher Scientific) with random primers (Toyobo Co., Ltd., Osaka, Japan). RT*–*PCR analysis was performed using an S1000 Thermal Cycler (Bio*–*Rad, Hercules, CA) with TaKaRa Ex Taq (TaKaRa, Shiga, Japan). Semiquantitative polymerase chain reaction (PCR) was performed for 25 cycles. Quantitative RT*–*PCR was performed using a TP870 Thermal Cycler Dice (TaKaRa) with Thunderbird SYBR qPCR Mix (TOYOBO CO., LTD. Osaka, Japan). The PCR primer pairs were as follows: mouse *Dcx*: 5′-GCTACATTTATACCATTGACGGATCCAG-3′ and 5′-TCATCACCAAAGAAATCATGGAGACAG-3′; mouse *Nestin*: 5′-GAGTCAGATCGCTCAGATCC-3′ and 5′-GGAGGACACCAGTAGAACTGG-3′; mouse *p35*: 5′-CTGCAGCCCATCCTCACATC-3′ and 5′-GAACACTTAAGTCTAGCGGTCGTTC-3′; mouse *Xbp-1*: 5′-GAATGCCCAAAAGGATATCAGACTC-3′ and 5′-GGCCTTGTGGTTGAGAACCAGGAG-3′; mouse *Dicer*: 5′-AGACCAACCTGCTCATTGCAAC-3′ and 5′-CACCATCCGCTGACTTCGAAC-3′; and mouse *β-Actin*: 5′-CCTAAGGCCAACCGTGAAAAG-3′ and 5′-CACGCACGATTTCCCTCTCA-3′.

### Statistics

Statistical analyses were performed using SPSS Statistics 27 (IBM, Armonk, NY) and Excel (Microsoft, Redmond, WA). Statistical significance (*p* < 0.05) was determined using Student’s t test (two-tailed) or a one-way ANOVA followed by the Bonferroni post-hoc test. The statistical details are summarized in Supplementary Table 2.

## Supplementary Information

Supplementary Information.

## Abbreviations

ATF4: Activating transcription factor 4
Aβ: Amyloid-β
eIF2α: Eukaryotic translation initiation factor 2α
ER: Endoplasmic reticulum
PBS: Phosphate-buffered saline
PCR: Polymerase chain reaction
siRNA: Small interfering RNA
UTR: Untranslated region
PKR: Protein kinase RNA-activated
PERK: PKR-like ER-localized eIF2α kinase
GCN2: General control nonderepressible 2
IRE1: Inositol-requiring protein-1
CHOP: C/EBP-homologous protein
