# Supplementary material for: Endoplasmic reticulum stress contributes to the decline in doublecortin expression in the immature neurons of mice with long-term obesity
Source: Sci Rep. 2022 Jan 19;12:1022. doi: 10.1038/s41598-022-05012-5 (PMC8770636; doi:10.1038/s41598-022-05012-5)
Supplement: Supplementary file 1 — Supplementary Information. [file 41598_2022_5012_MOESM1_ESM.docx]

**Supplementary Figure legends**

**Supple. Fig. 1** ***Performance of db/db mice in the MWM test***

**(a)** Body weights (BWs) of *db/db* mice. Control mice (n=5-7); *db/db* mice (n=5-7). **(b)** Performance of *db/db* mice in the MWM test. Control mice (n=8); *db/db* mice (n=8). The total distance traveled (arbitrary units) and speed (arbitrary units) as well as the latency to reach the invisible platform over four days are shown. Repeated measures ANOVA (genotype × day) indicated that the escape latencies differed significantly between control and *db/db* mice [degree of freedom: 1 (genotype), 14 (errors); F-score: 7.655; *p*=0.015]; however, the escape latencies of control mice did not differ between days 1 and 4 (*p*=0.174). The numbers of platform crossings during the probe test on day 5 are shown. Statistical significance (*p* < 0.05) was determined by Student’s t test. *: *p<0.05*. The error bars represent the standard errors of the mean (SEMs). n.s.: not significant.

**Supple. Fig. 2** ***Memory was impaired in aged C57BL/6 mice with long-term obesity***

**(a)** The body weights (BWs) of C57BL/6 (n=12 standard diet-fed mice; n=12 HFD-fed mice) mice. C57BL/6 mice (10 weeks old) were fed a standard chow diet (st.) or a HFD (60% fat) for 67 weeks. The error bars represent the SEMs. *: *p<0.05*. Statistical significance (*p* < 0.05) was determined by Student’s t test. **(b)** Serum levels of FBS and insulin in standard diet-fed mice (n=12) and HFD-fed mice (n=12). The error bars represent the SEMs. Statistical significance (*p* < 0.05) was determined by Student’s t test. **(c)** NOL protocol. Each mouse was habituated to a cage without objects for 15 min on day 1. The mice were exposed to three different objects, i.e., conical (diameter x height: 5 x 11.5 cm), cylindrical (6.5 x 10.5 cm), and reagent (5 x 13.2 cm) bottles, for 5 min three times at 2-min intervals on day 2. On day 3, the mice were exposed to three objects for 5 min three times at 2-min intervals and placed in their home cages for a retention interval (two minutes). Then, one of the objects was moved to the opposite corner. The behavior of each mouse was monitored using video recording software and an automated tracking system. (**d**) Representative images of mice exploring the objects in trials 3 and 4 (left panels). The area was divided into four zones (2, 3, P, and E1 at trial3; 2, 3, N, and E2 at trial4). The objects (circle) were placed in zones 2, 3 or P in trial 3 and in zones 2, 3 or N in trial 4. The behaviors of the mice in each zone were monitored using video recording software and an automated tracking system (SMART v3.0 software; Panlab, Barcelona, Spain). Representative trajectories of mice exploring the objects at trial3 and trial4 (standard diet-fed mice: upper panels; HFD-fed mice: lower panels) (middle panels). Total distance traveled (arbitrary units: A. U.) (right panels). Statistical significance (*p* < 0.05) was determined by Student’s t test. **(e)** The exploration indices in each zone in trials 3 and 4. The exploration index was calculated as the time spent exploring the object placed in one zone divided by the total time spent in all four zones [index P: P/(2 + 3 + P + E1); index N: N/(2 + 3 + N + E2); index 2: 2/(2 + 3 + P + E1 or 2 + 3 + N + E2); index 3: 3/(2 + 3 + P + E1 or 2 + 3 + N + E2)]. Statistical significance (*p* < 0.05) was determined by Student’s t test when comparing zones P and N and by one-way ANOVA followed by the Bonferroni post-hoc test for at trials 3 and 4. Standard diet-fed mice in trial 3: F(3,44)=3.88, p<0.02; in trial 4: F(3,44)=16.8, p<0.05. HFD diet-fed mice in trial 3: F(3,36)=2.68, p=0.06; in trial 4: F(3,36)=2.14, p=0.11. Standard diet-fed mice (n=12); HFD-fed mice (n=10).

**Supple. Fig. 3** ***The ER stress was activated in the brains of C57BL/6 mice with long-term obesity***

CHOP and ATF4 expression in the hippocampi of 86-week-old C57BL/6 mice with long-term HFD-induced obesity. Mice were fed a HFD for 74 weeks. st.: standard diet; H: high-fat diet. The arrow indicates ATF4, and the arrowhead indicates Dcx. Statistical significance (*p* < 0.05) was determined by Student’s t test.

**Supple. Fig. 4 *Categorization of the dendritic morphologies of Dcx-positive cells in the DGs of db/db mice at the age of 45 weeks old***

Dcx-positive cells from 45-week-old control (mouse 1: 105 Dcx-positive cells; mouse 2: 126 Dcx-positive cells) and *db/db* (mouse 1: 96 Dcx-positive cells; mouse 2: 62 Dcx-positive cells) mice were categorized. Plumpe, T. *et al* characterized six categories of Dcx-positive cells using dendritic morphology analysis as follows: A: no processes; B: process length less than one nucleus-wide (< 10 μm); C: process length longer than in B; D: process reaching molecular layer; E: one strong dendrite branching in the molecular layer; and F: delicate dendritic tree branching in the granule cell layer.


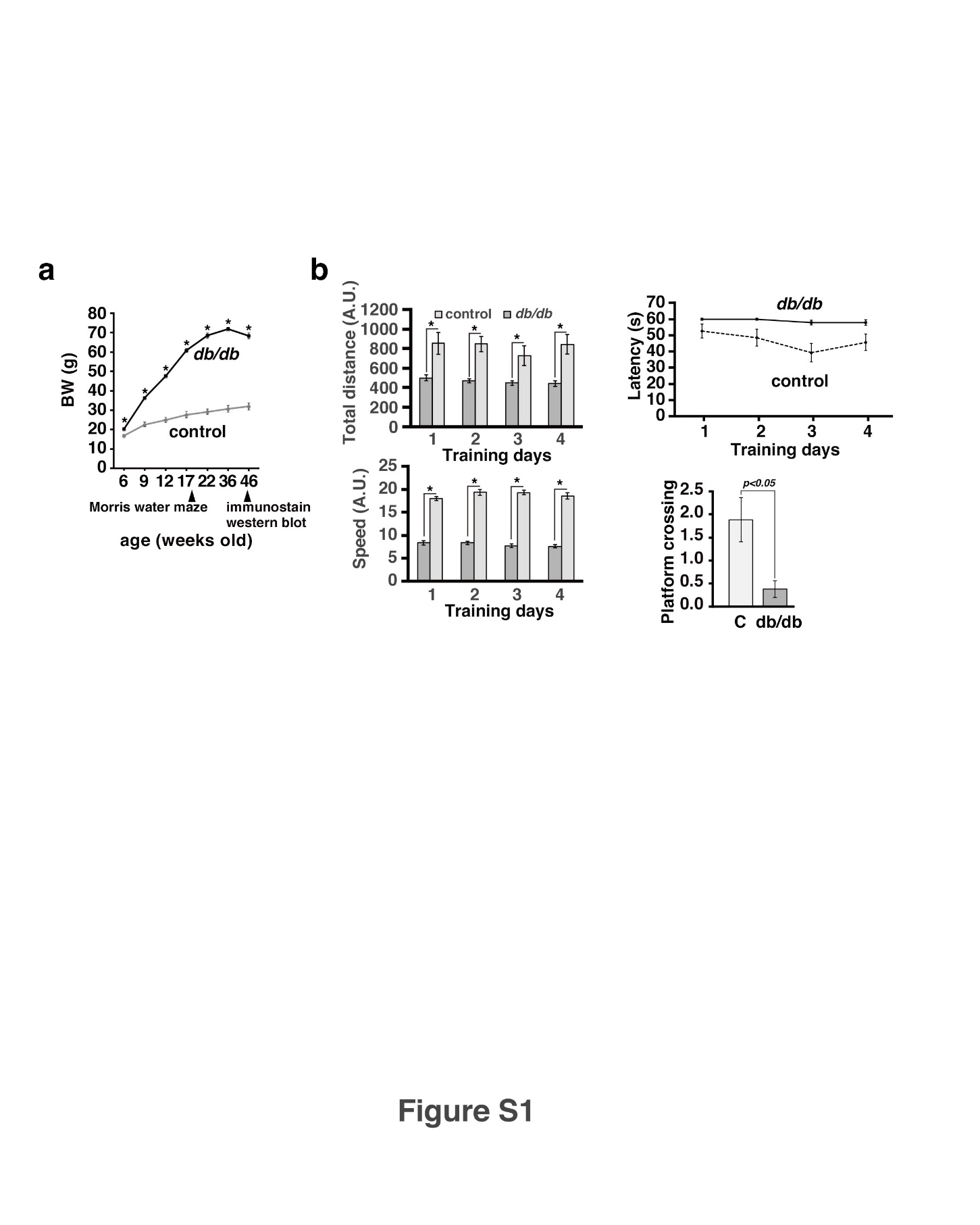


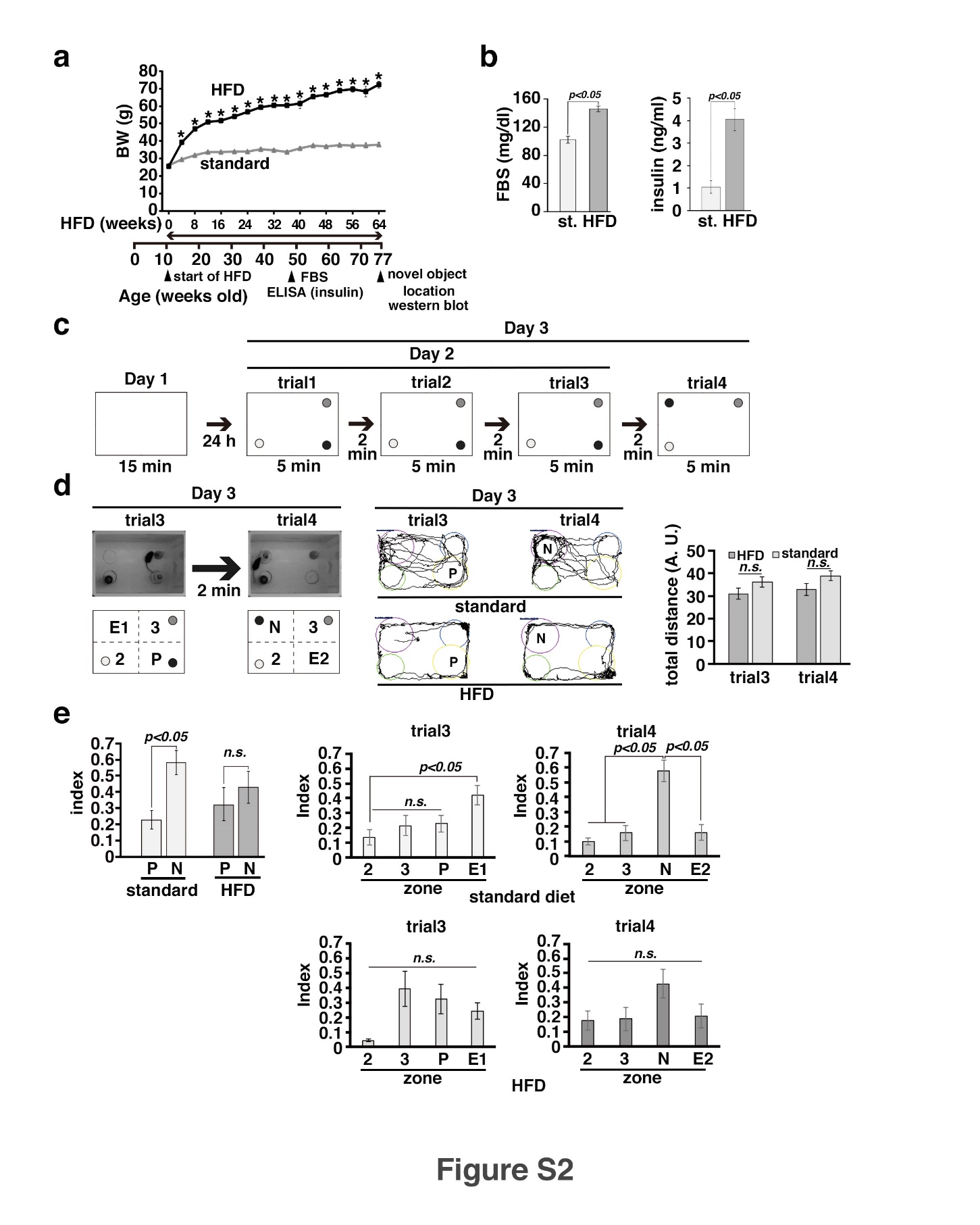


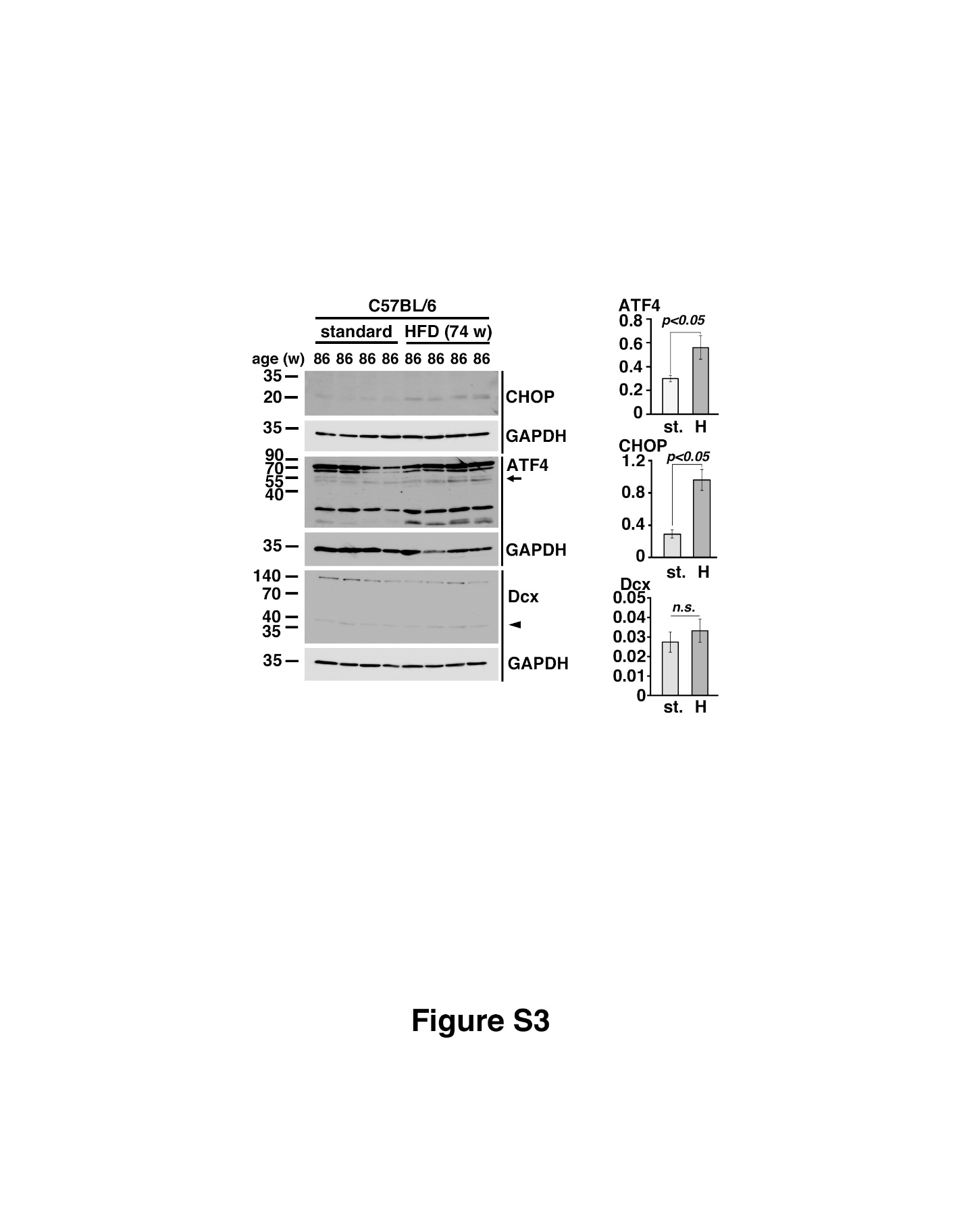


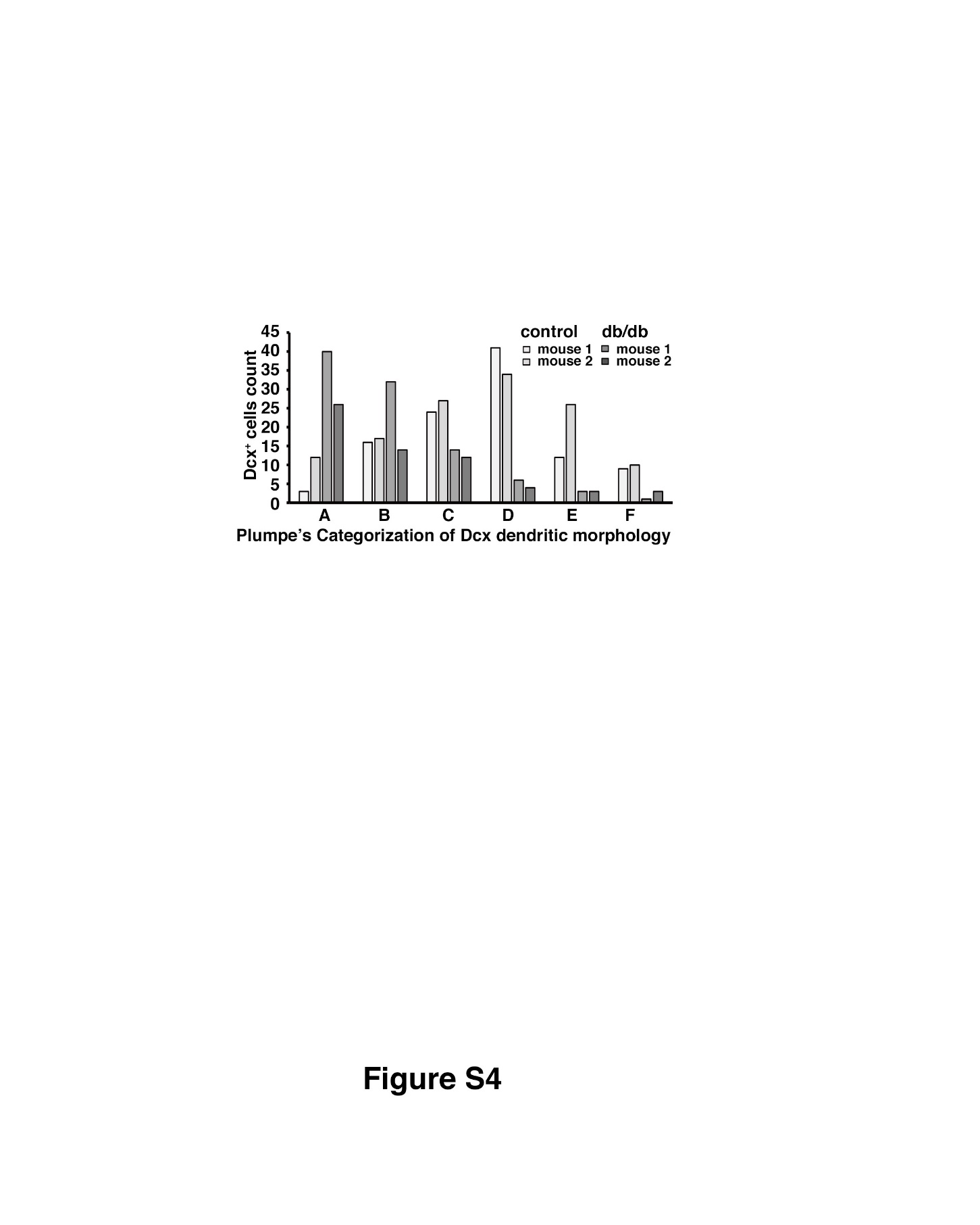


**Supplementary Tables**

**Supple. Table 1** ***Information regarding the age-protocol-strain-experiment***


**Supple. Table 2** ***Statistical summary***
